# GluN2D-mediated excitatory drive onto medial prefrontal cortical PV+ fast-spiking inhibitory interneurons

**DOI:** 10.1371/journal.pone.0233895

**Published:** 2020-06-04

**Authors:** Jonathan Garst-Orozco, Ruchi Malik, Thomas A. Lanz, Mark L. Weber, Hualin Xi, Dominique Arion, John F. Enwright, David A. Lewis, Patricio O’Donnell, Vikaas S. Sohal, Derek L. Buhl

**Affiliations:** 1 Pfizer Neuroscience Research Unit, Cambridge, Massachusetts, United States of America; 2 Department of Psychiatry and UCSF Weill Institute for Neurosciences, San Francisco, California, United States of America; 3 Center for Integrative Neuroscience, University of California San Francisco, San Francisco, California, United States of America; 4 Sloan-Swartz Center for Theoretical Neurobiology, University of California San Francisco, San Francisco, California, United States of America; 5 Department of Psychiatry, University of Pittsburgh, Pittsburgh, Pennsylvania, United States of America; 6 Department of Neuroscience, University of Pittsburgh, Pittsburgh, Pennsylvania, United States of America; University of Kentucky, UNITED STATES

## Abstract

Deficits in fast-spiking inhibitory interneurons (FSINs) within the dorsolateral prefrontal cortex (dlPFC) are hypothesized to underlie cognitive impairment associated with schizophrenia. Though representing a minority of interneurons, this key cell type coordinates broad neural network gamma-frequency oscillations, associated with cognition and cognitive flexibility. Here we report expression of GluN2D mRNA selectively in parvalbumin positive cells of human postmortem dlPFC tissue, but not pyramidal neurons, with little to no GluN2C expression in either cell type. In acute murine mPFC slices the GluN2C/D selective positive allosteric modulator (PAM), CIQ(+), increased the intrinsic excitability as well as enhanced NMDAR-mediated EPSCs onto FSINs. This increase in intrinsic excitability with GluN2C/D PAM was also observed in the Dlx 5/6+/- FSIN developmental deficit model with reported FSIN hypoexcitability. Together these data speak to selective modulation of FSINs by a GluN2D PAM, providing a potential mechanism to counter the FSIN-deficit seen in schizophrenia.

## Introduction

Studies of the underlying pathophysiology of schizophrenia have led to the NMDA, GABA and dopaminergic hypotheses, backed by pharmacological manipulations that replicate key aspects of the pathology. The dopamine (DA) hypothesis, which has remained the most prevalent in the field, is substantiated by the ability of DA agonists to replicate, and DA antagonists to address, positive symptoms. Though positive symptoms are perhaps the most striking aspect of the disease, negative and cognitive symptoms have been shown to be more predictive of patient outcome and remain unaddressed by current pharmacology [1–5]. These deficits include working memory, attention, cognitive flexibility, semantic processing and verbal learning. In addition to the cognitive impairment associated with schizophrenia (CIAS), these subjects also exhibit an associated marked decrease in evoked cortical gamma band frequency (30–80 Hz) amplitude, which is thought to be indicative of fast-spiking interneuron (FSIN) dysfunction in synchronizing the firing of broad neural networks [6–9]. The GABA hypothesis is also supported by human post-mortem tissue from individuals with schizophrenia showing markedly reduced GAD67 expression [10,11], an enzyme that synthesizes GABA, in addition to reduced GAT1 [12], the membrane transporter of GABA, somatostatin (SOM) [13] and parvalbumin (PV) [14], a calcium buffer expressed within FSINs in the dorsolateral prefrontal cortex (dlPFC). The NMDA hypothesis stems from the ability of NMDAR antagonists phencyclidine (PCP) and ketamine to induce both positive and cognitive deficits in healthy subjects that mirror schizophrenia and reinstate symptoms in stabilized patients with schizophrenia [15]. Accompanying the induction of cognitive deficits is a marked increase in neuronal firing and decreased coordinated bursting within the PFC [16], suggesting NMDAR antagonists act with greatest efficacy at inhibitory interneurons [17].

One possible site of convergence of the glutamatergic, GABAergic and dopaminergic hypotheses is at the level of inhibitory interneurons, which are driven by NMDA-mediated excitatory inputs and control inhibition of the excitatory pyramidal network throughout the brain, including in the hippocampus, which drives downstream DA neurons [18]. The nexus of the hypotheses at inhibitory interneurons offers a target for therapeutic intervention in selectively boosting inhibition [19]. Previous data point to the selective expression of GluN2C and GluN2D receptors at key classes of interneurons in the adult rodent hippocampus [20]. Though initially expressed in both pyramidal and inhibitory interneurons at birth, GluN2D expression decreases with development, becoming selectively enriched within PV- and somatostatin (SOM)-expressing fast-spiking interneurons in the adult hippocampus [21]. GluN2D expression was confirmed functionally by the modulation of NMDAR-mediated current using CIQ(+), a GluN2C/D specific PAM [22], in hippocampal interneurons of young mice, but not in pyramidal cells [23], nor in the GluN2D -/- animals [24].

Here we report that patterns of GluN2D selective expression described in the young murine hippocampus are conserved in PV+ interneurons of the dlPFC in postmortem adult human tissue. Consistent with previous findings, we show CIQ(+) increases the FSIN intrinsic excitability in addition to potentiating NMDAR-mediated excitatory currents onto FSINs in the adult mPFC. Together these results point to the viability of GluN2D-selective pharmacology in the remediation of NMDAR- and GABAergic hypofunction in schizophrenia.

## Materials and methods

### Human transcriptomics

#### Human laser-capture microdissection

Post-mortem brain samples were obtained from human subjects with a relatively small spread in age, low post-mortem interval (PMI) and limited agonal state to ensure high quality RNA could be collected. Subjects had no known neuropsychiatric or neurodegenerative disorders. No statistically significant differences between the two groups were found for age, PMI, brain pH, RIN or storage time (summarized in Table 1). Laser-capture methodology for RNA has been described previously [25,26]. From each sample, 12 mm sections of dlPFC (Brodmann area 9) were cut and stained with thionin for pyramidal neurons, or aggrecan for parvalbumin interneurons. For each cell type, 200 cells were cut from deep layer 3 and collected into RLT buffer with β-mercaptoethanol, then stored at -80°C. Adjacent stained and unstained slides were collected in the same manner to serve as controls. Informed consent for brain tissue donation was obtained from the next-of-kin via a recorded and witnessed telephone call with a licensed clinician using procedures approved by the University of Pittsburgh Committee on Research and Clinical Training Involving Decedents.

**Table 1:** Human subject and sample information.

Characteristic	Microarray	RNAseq
Number	7	4
Sex	3M, 4F	4M
Race	7W	2 W, 2 B
Age, years	48.1 (6.6)	41.5 (7.7)
PMI, hours	19.7 (6.6)	19.3 (9.8)
Brain pH	6.7 (0.2)	6.7 (0.2)
RIN	8.0 (0.7)	8.0 (0.8)
Storage time	121.5 (38)	146.3 (48)

Values represent number or mean (SD); W/B represent white/black; PMI = post-mortem interval; RIN = RNA integrity number; storage time is given in months at -80°C.

#### Human transcriptional profiling

Generation of microarray data from pyramidal and PV neurons was previously described [25,26]. For RNAseq, RNA was isolated using RNeasy micro kits (Qiagen), and cDNA libraries were prepared using the pico input SMARTer stranded total RNA-seq kit (Clontech). Library size was measured by high sensitivity DNA kit (Agilent), and concentration was quantified by Qubit (Life Technologies). Libraries were sequenced on a NextSeq500 (Illumina), and reads were aligned to the human genome using SALMON [27], and differential expression of genes between cell types was quantified using DESEQ2. On average, 5 million reads mapped to annotated gene regions for each sample. The number of genes with TPM (transcripts per million) >1 was 12,000–13,000 for the isolated cell types, and over 16,000 for slide controls. All data have been deposited to GEO (GSE149154).

### Murine electrophysiology

All procedures were approved by the Institutional Animal Care and Use Committees of the Pfizer Neuroscience Research Unit and the University of California, San Francisco.

Adult (> 8-week old) male GAD67-GFP C57BL/6 mice (G42 line, Jackson Labs) were anesthetized with inhaled isoflurane and transcardially perfused with oxygenated ice-cold aCSF cutting solution containing (in mM): 130 NaCl, 26 NaHCO_3_, 2.5 KCl, 1.25 NaH_2_PO_4_, 0.5 CaCl_2_, 3 MgSO_4_ and 10 glucose. Adult (P40-P60) Dlx 5/6^+/^ mice (see [28]) were anesthetized with an intraperitoneal injection of euthasol and transcardially perfused with oxygenated ice-cold cutting solution containing (in mM): 210 sucrose, 2.5 KCl, 1.25 NaH_2_PO_4_, 25 NaHCO_3_, 0.5 CaCl_2_, 7 MgCl_2_ and 7 dextrose. Following rapid decapitation, brains were removed and sectioned coronally on a vibrating microtome (VT1200S, Leica Biosystems). 250-μm slices containing the mPFC were transferred to warm (35°C) oxygenated aCSF containing (in mM) 130 NaCl, 26 NaHCO_3_, 2.5 KCl, 1.25 NaH_2_PO_4_, 2.5 CaCl_2_, 1 MgSO_4_ and 10 glucose and allowed to recover for > 1hr. Slices were then transferred to a recording chamber with aCSF containing 50 *μ*M picrotoxin and 10 *μ*M CNQX (Sigma Aldrich) heated to 35°C. Whole-cell recordings were performed on layer 5/6 neurons using (4–8 MOhm) electrodes containing (in mM): 150 K-Gluconate, 10 HEPES, 5 NaCl, 1 MgCl, 0.2 EGTA, 2 Mg-ATP and 0.5 Na-GTP. Cells were characterized in current clamp in response to 500 ms DC injections from –100 to +300 pA in 50 pA steps. Inhibitory (GFP+) FSINs were identified by rapid (>200 Hz) sustained firing [29]. Pyramidal cells with large (GFP-) somata showed rapid spike-frequency adaptation. For EPSC measures neurons were held at –50 mV to characterize weakly rectifying GluN2D-mediated evoked currents in response to bipolar stimulation of layer 2/3. Stimulation intensity was set to consistently evoke an EPSC with stable current amplitude. CIQ was dissolved in DMSO stock prior to dilution 1/1000. Baseline EPSCs were recorded for 10 min in solution containing DMSO vehicle prior to application of solution containing 10 μM CIQ(+) for 15 min. Average current amplitude for the final 5 min period was compared to that of the 5 minutes prior to application of CIQ(+). During current clamp recordings, series resistance and pipette capacitance were appropriately compensated. Series resistance was usually 10–20 MΩ, and experiments were terminated if series resistance exceeded 25 MΩ.

### Data analysis

Data were analyzed using custom routines written in IGOR Pro (Wavemetrics). Input resistance was calculated from the steady-state current in response to a 10 mV step for voltage-clamp experiments and from the slope of the linear fit of the voltage–current plot generated from a family of hyperpolarizing and depolarizing current injections (-50 to +20 pA, steps of 10 pA) for current-clamp experiments. The membrane time constant (tau) was calculated as the slow component of a double‐exponential fit of the average voltage decay in response to a hyperpolarizing current injection (−400 pA, 1 ms). Firing output was calculated as the number of action potentials (APs) fired in response to 800 ms long depolarizing current injections (25–300 pA). Firing frequency was calculated as the number of APs fired per second. Sigmoid fits of firing frequency–current curves were used to obtain xhalf and linear rising rates. Rheobase was measured as the minimum current injection that elicited spiking. Firing traces in response to 50 pA current above the rheobase were used for analysis of single AP properties–AP threshold, maximum *dV*/*dt* (rate of rise of AP), AP amplitude, AP half-width and fast after hyperpolarization (fAHP) amplitude. Threshold was defined as the voltage at which the value of third derivative of voltage with time is maximal. Action potential amplitude was measured from threshold to peak, with the half-width measured at half this distance. Fast after hyperpolarization (fAHP) was measured from the threshold to the negative voltage peak after the AP.

## Results

### GluN2D transcription is selectively enriched in PV+ interneurons of the human dlPFC

**Fig 1:**
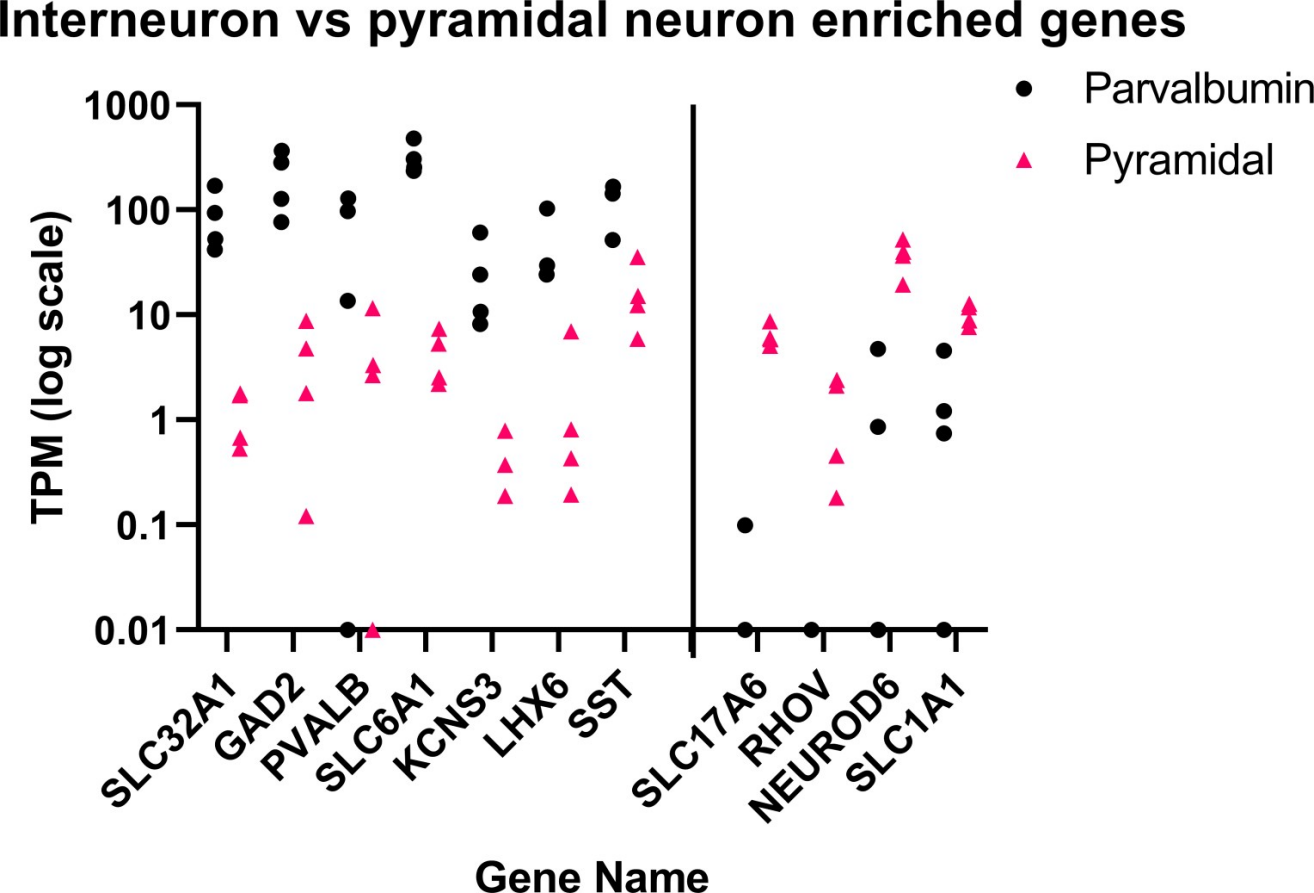
Interneuron versus pyramidal neuron enriched genes. Expression of cell-type specific or enriched genes in parvalbumin interneurons (black circles) versus pyramidal neurons (red triangles). Each point represents 200 laser-captured cells pooled from a single brain section. The y-axis represents TPM expression of each gene on a log scale. A vertical line separates interneuron markers (left) from pyramidal neuron markers (right).

**Fig 2:**
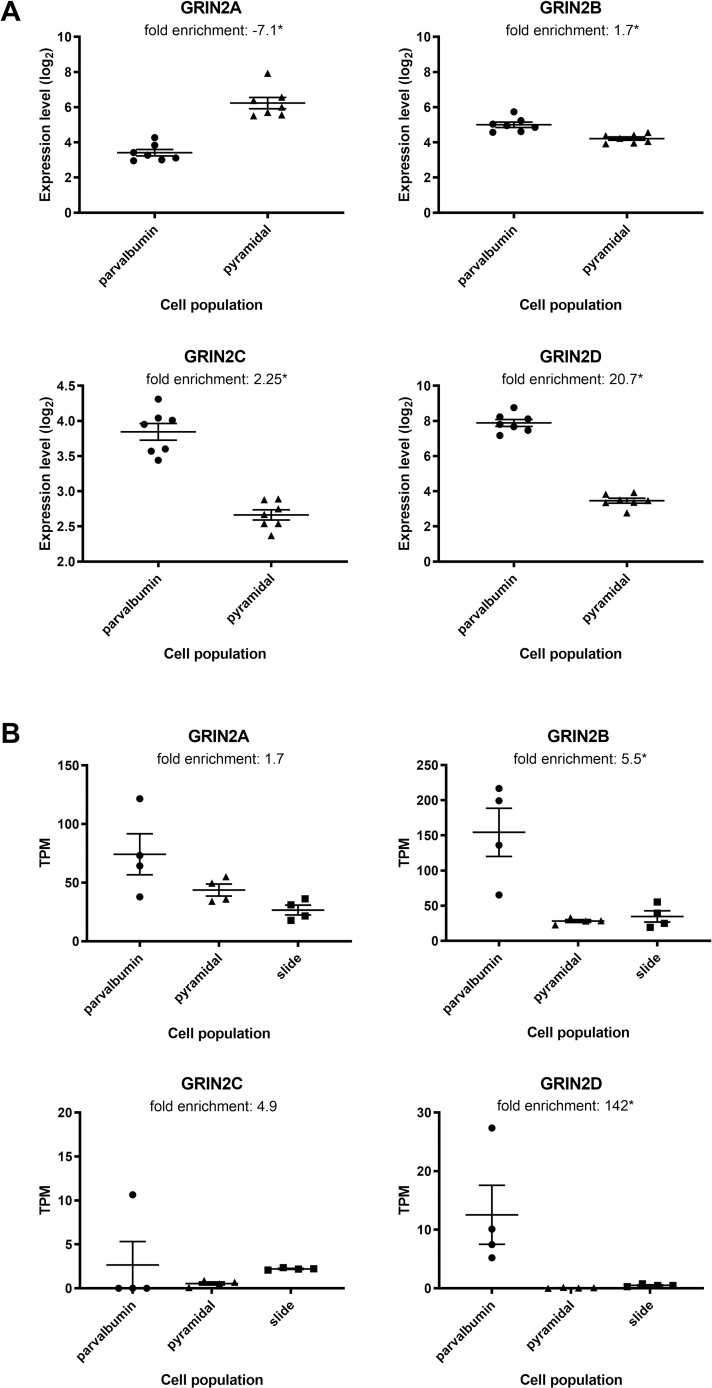
GluN2D transcripts are enriched in PV interneurons relative to pyramidal neurons in the dlPFC. (A) Gene expression data as measured by microarray is plotted for GluN2A, B, C and D, and enrichment in one cell type versus the other is listed. Triangles represent pyramidal neurons, circles represent parvalbumin interneurons; intensity is expressed on a log2 scale. *p<0.05 for parvalbumin versus pyramidal neurons. (B) Gene expression data measured from a separate cohort of samples by RNAseq is presented for pyramidal neurons (triangles), parvalbumin interneurons (circles), and adjacent sections on LCM slides (squares). Fold enrichment between parvalbumin neurons and pyramidal neurons is listed above each graph. The y-axis represents the absolute value of gene expression as TPM. *p<0.05 for parvalbumin versus pyramidal neurons.

Laser-capture microdissection was performed on thionin-stained pyramidal neurons and aggrecan-stained parvalbumin neurons in dlPFC. For each cell type, 100–200 microdissected cells were collected and processed for RNAseq alongside stained adjacent gray matter sections. Fig 1 demonstrates selectivity of each cell population for markers characteristic of each neuron type, such as VGAT (SLC32A1) and VGLUT2 (SLC17A6). Expression of the GluN2 subunits is shown in Fig 2 for the RNAseq samples, along with a second set of samples processed for microarray. GluN2A and B were expressed in both pyramidal neurons and PV interneurons, but also showed expression in the adjacent sections, suggesting that expression is not enriched in pyramidal or PV neurons relative to other cell types present in cortical gray matter. GluN2C showed very low expression in pyramidal neurons and parvalbumin interneurons, and higher expression in the section, suggesting that the majority of GluN2C expression in these regions comes from another cell type. GluN2D showed a clear enrichment in microdissected parvalbumin interneurons versus pyramidal neurons in both microarray and RNAseq data. The relatively low level of GluN2D in full sections relative to parvalbumin interneurons suggests that the latter cell type is the predominant cell expressing this receptor subtype in dlPFC. The interneurons stained with aggrecan expressed other markers of PV+ interneurons, such as KCNS3, but also some markers common to both PV+ and SST, such as LHX6 and SST, thus expression should be interneuron-specific, but not completely restricted to PV+ interneurons. The full TPM table for all samples can be found in S1 Table.

### GluN2C/D PAM selectively enhances both FSIN intrinsic excitability and NMDAR-mediated excitatory drive onto FSINs

To test the functional significance of modulating GluN2D receptors we moved to murine ex-vivo acute brain slices containing the mPFC from adult GAD67-GFP mice, showing selective GFP expression in FSINs [29]. Layer 5/6 neurons were recorded using whole-cell voltage and current clamp to quantify the excitatory drive onto the cell and neuronal intrinsic excitability, respectively. The effect of GluN2C/D PAM CIQ(+) 10 μM was evaluated within cells before and after bath application [24]. FSINs were identified by GFP fluorescence soma and functionally confirmed by DC-evoked spiking at characteristic high rates >200 Hz with minimal spike-frequency adaptation (Fig 2A) [30]. Pyramidal neurons were identified by large GFP(-) soma and pronounced spike-frequency adaptation.

**Fig 3:**
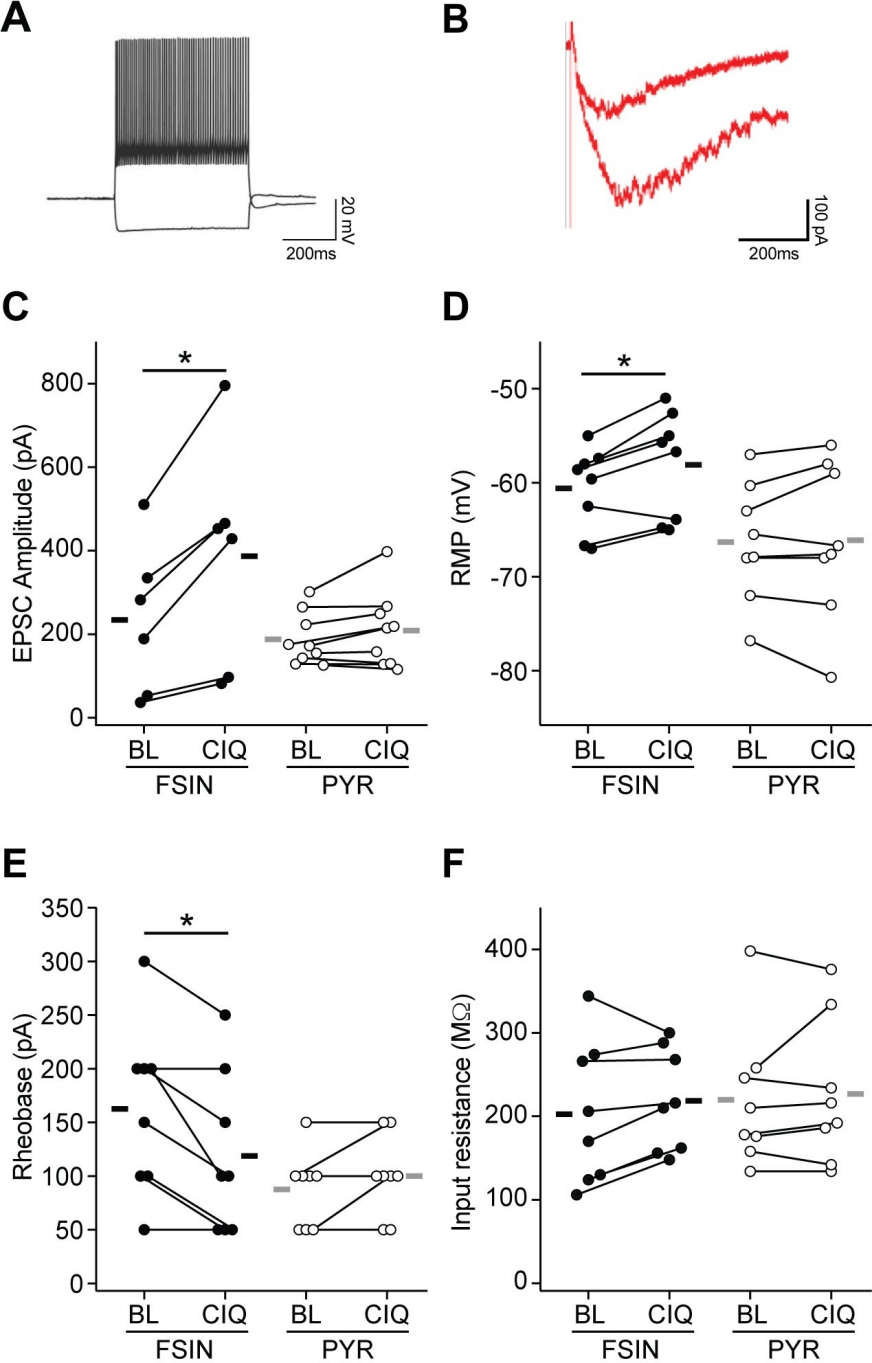
GluN2C/D PAM CIQ(+) increased excitatory drive and intrinsic excitability of fast-spiking interneurons in the mPFC of adult mice. (A) GFP(+) FSINs showed characteristic sustained high-frequency firing. (B) Sample NMDAR-mediated EPSC onto FSIN was potentiated upon the addition of CIQ(+) 10 μM. (C) EPSC potentiation was seen in FSINs (average 83% increase, p = 0.017, two-way paired t-tests), but not excitatory pyramidal neurons (p = 0.55, two-way paired t-tests). n = 6 FSIN from 6 mice, 9 Pyramidal from 7 mice. (D) CIQ depolarized the resting membrane potential of FSINs (average +2.5mV, p = 0.039, two-way paired t-tests) but not pyramidal neurons (p = 0.84, two-way paired t-tests). n = 8 FSIN from 7 mice, 8 Pyramidal from 6 mice. (E) CIQ increased the FSIN excitability seen in a reduction in rheobase (p = 0.041, two-way paired t-tests). Effect was not seen in pyramidal neurons (p = 0.17, two-way paired t-tests). n = 8 FSIN from 7 mice, 8 Pyramidal from 6 mice. (F) Application of CIQ did not affect the input resistance of FSIN (p = 0.19, two-way paired t-tests) or pyramidal neurons (p = 0.63, two-way paired t-tests). n = 8 FSIN from 7 mice, 8 Pyramidal from 6 mice. *p<0.05; BL = Baseline.

NMDAR-mediated evoked ESPCs were isolated by bath application of picrotoxin and CNQX to block GABAR- and AMPA-mediated currents. EPSCs were evoked with a stimulating electrode placed in layer 2/3. Neurons were held at -50 mV to enhance currents from weakly rectifying GluN2D receptors. EPSCs from FSINs were significantly potentiated upon the addition of 10 μM CIQ(+), an effect not seen in neighboring pyramidal neurons (Fig 3B and 3C). Current-clamp recording revealed a significantly enhanced intrinsic excitability upon the addition of CIQ, seen in a depolarized resting membrane potential and a decrease in rheobase in FSINs but not pyramidal neurons (Fig 3D and 3E). No change in input resistance was detected upon the addition of CIQ in either FSINs or pyramidal neurons (Fig 3F). Together these experiments demonstrate modulation of GluN2C/D receptors selectively increase both excitatory drive onto and intrinsic excitability of FSINs.

### GluN2C/D PAM reverses hypoexcitability in Dlx5/6+/- FSIN deficit model

**Fig 4:**
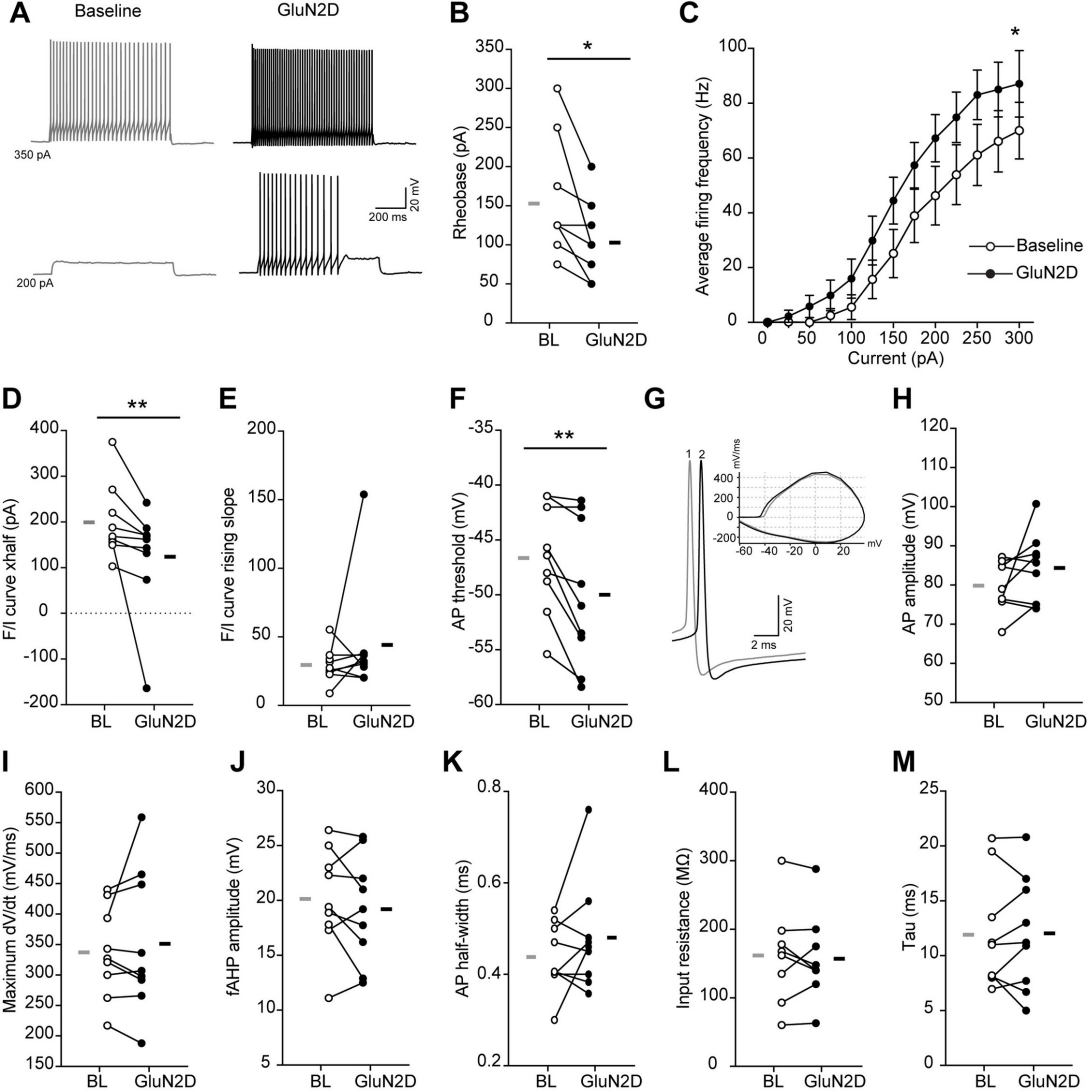
GluN2C/D PAM CIQ(+) increased intrinsic excitability of FSINs in Dlx 5/6^+/-^ mice. (A) Example voltage traces showing the firing output in response to depolarizing direct current injections in a FSIN during baseline and after application of CIQ(+) 10mM. (B) CIQ(+) application decreases the rheobase of FSINs (t_8_ = 3.09, p = 0.01, two-way paired t-tests). (C) Average firing frequency in response to depolarizing current steps is compared for baseline and after CIQ(+) application (Effect of treatment: F_(1,8)_ = 6.08, p = 0.03, two-way repeated measures ANOVA). (D-E) Frequency-current (F/I) curves of FSINs during baseline and after drug application were fitted with sigmoids. Xhalf (p = 0.004, Wilcoxon rank test) and rising slope (p = 0.09, Wilcoxon rank test) of the F/I sigmoids fits are compared in D and E, respectively. (F) Application of CIQ(+) decreases the AP threshold (t_8_ = 3.6, p = 0.006, two-way paired t-tests). (G) Example voltage traces showing single action potentials (APs) recorded during baseline (1) and after application of CIQ(+) (2). Inset, phase-plane plots of the example APs. (H-K) Application of CIQ(+) does not affect AP amplitude (t_8_ = 1.6, p = 0.14, two-way paired t-tests), maximum rate of rise (dV/dt; t_8_ = 0.68, p = 0.51, two-way paired t-tests), fAHP amplitude (t_8_ = 1.05, p = 0.32, two-way paired t-tests), and half-width (t_8_ = 1.34, p = 0.1, two-way paired t-tests). (L-M) Application of CIQ(+) does not affect the input resistance (t_8_ = 0.55, p = 0.59, two-way paired t-tests) and membrane time constant (tau; t_8_ = 0.17, p = 0.86, two-way paired t-tests) of FSINs. N = 9 cells from 3 mice; * P<0.05, ** P<0.01; BL = Baseline.

Postmortem work from individuals with schizophrenia show deficits in markers of fast-spiking interneurons, leaving open the question of whether GluN2D modulation remains a viable therapy in the disease-compromised state. The Dlx5/6^+/-^ mouse model shows an age-dependent deficit in PV interneuron function including hypoexcitability, increased input resistance, slower membrane time constant and prolonged action potential half-width, accompanying deficits in task-evoked gamma oscillations and cognitive deficits seen in schizophrenia [7,28]. To assess whether GluN2D pharmacology shows efficacy in reversing FSIN deficits in an FSIN-compromised state, we evaluated the effect of CIQ(+) on subthreshold and firing properties in FSINs from adult Dlx 5/6^+/-^ mice. These FSINs showed a robust increase in intrinsic excitability in response to brief (15 min) CIQ(+) application (Fig 4A), seen in a reduced rheobase (Fig 4B) and leftward shift in the F-I curve (Fig 4C and 4D). No change was observed in the slope of the F-I curve (Fig 4E), suggesting an effect mediated by changes in neuron action potential threshold, rather than a change in the F-I dynamic range. This was confirmed by the reduction in action potential threshold in response to a voltage ramp (Fig 4F). No significant change was observed in spike waveform (Fig 4G–4K) or in passive properties of the neuron, namely input resistance and membrane time constant (Fig 4L and 4M) upon the addition of CIQ. Overall, these findings highlight that GluN2C/D PAM increases the intrinsic excitability of FSIN in Dlx5/6^+/-^ mice by lowering the voltage and current threshold for firing.

## Discussion

Previous work in post-mortem brains from patients with schizophrenia have found lower expression of parvalbumin and GAD67 mRNAs in cases relative to age-matched controls [10,11,14]. Reduced expression of GAD67 protein has been found in parvalbumin interneuron terminals [31], and a reduction in mean parvalbumin intensity has been measured in parvalbumin interneurons in schizophrenia without an alteration in PV neuron density [32]. These findings suggest that the number of parvalbumin interneurons is not lower in schizophrenia, but rather that they have either an intrinsic deficit or down-regulated expression of genes central to the function of these neurons in response to an upstream insult.

Reductions in parvalbumin, with accompanying disruptions in cortico-hippocampal circuitry, have also been found in a number of neurodevelopmental animal models. These models include genetic risk factors such as a DISC1 truncation or murine version of the 22q11 microdeletion [33,34] and in utero exposure to teratogens (e.g. methylazoxymethanol acetate) or inflammation (e.g. response to poly I:C) [35,36]. Further, preclinical studies have demonstrated that modulation of PV+ FSINs rescue certain cognitive deficits associated with schizophrenia [7]. The convergence of different developmental mechanisms on a pathological finding observed in schizophrenia post-mortem brain make the modulation of parvalbumin-containing FSINs an attractive potential therapeutic target.

Glutamate acting on AMPA receptors is a primary driver of FSIN and pyramidal neuron activity, however, we are aware of no reports of differential expression in FSINs in the adult brain. Our electrophysiological data show GluN2C/D functional modulation in FSINs, but not pyramidal neurons, within the murine prefrontal cortex. Our findings demonstrate that both GluN2C/D-mediated potentiated excitatory drive (EPSCs) and enhanced intrinsic excitability act synergistically to increase the output of this neuronal functional class. The addition of CIQ(+) depolarized the resting membrane potential of FSINs, suggesting GluN2C/D-containing receptors are active at rest, as has been reported in interneurons of the developing cortex [37]. This unique feature of GluN2C/D-containing NMDARs can be attributed to basal glutamatergic tone acting with higher potency (~0.5 *μ*M) at GluN2D-containing NMDARs, which are active at rest due to weak Mg^2+^-induced rectification [20,38]. This tonic activity has been shown to be developmentally regulated, decreasing together with GluN2D expression. However, selective refined expression onto FSINs would likely leave them sensitive to pharmacological GluN2C/D-mediated potentiation of excitatory drive and intrinsic excitability even in the adult brain. Additional experiments in the presence of selective NMDAR antagonists would test whether this effect is mediated directly by potentiation of NMDAR tonic signaling.

Our work in human and murine tissue demonstrate a conserved selective expression of GluN2D in FSINs of the mature prefrontal cortex, a key functional class of cells implicated in cognitive impairments associated with schizophrenia (CIAS). The selective transcription we report in human dlPFC PV+ interneurons presents a target for specific potentiation as a means to reverse the FSIN deficit and ensuing impaired cognitive performance. No difference in GluN2D expression was found in PV interneurons between schizophrenia subjects and healthy controls in a previously published microarray dataset [26]. The idea that GluN2D could be a viable therapeutic target is also supported by previous reports in Dlx5/6^+/-^ mice, in which mPFC FSINs exhibited decreased functional properties that were associated with cognitive deficits *in vivo*, which were reversed with optogenetic stimulation of mPFC FSINs [7]. These findings suggest that selective modulation of FSINs, perhaps via pharmacological GluN2D potentiation, could present a viable procognitive therapeutic intervention to remedy interneuron hypofunction underlying CIAS. The generation of selective GluN2D PAMs would curtail potential side effects mediated by high GluN2C expression within the mature cerebellum [20].

Our data offer a mechanism by which FSINs may drive cognitive impairments and altered gamma frequency oscillations induced by NMDAR antagonists ketamine or PCP. Inhibition of GluN2D-mediated excitatory drive onto FSIN and downstream disinhibition may contribute to cognitive disruptions seen with ketamine, an effect absent in GluN2D KO mice [39]. Interestingly, PCP-induced hyperlocomotion and enhanced dopamine release are also absent in GluN2D KO animals [40], suggesting GluN2D may also contribute to positive symptoms in schizophrenia via expression in the ventral tegmental area or basal ganglia [20,41].

Our data support the hypothesis that GluN2D modulation may be a viable therapeutic to remediate FSIN hypofunction in disease-compromised states. Our therapeutic hypothesis is further supported by our functional data from Dlx5/6^+/-^ mice, showing GluN2D potentiation reverses FSIN hypoexcitability [7]. GluN2D potentiation, however, did not acutely reverse deficits in passive membrane properties, namely increased input resistance and slower membrane time constant, effects that may be driven by longer term homeostatic plasticity to compensate for FSIN hypoexcitability in Dlx5/6+/- mice. The effect of boosting FSIN excitability with GluN2D positive allosteric modulation on task evoked gamma and cognitive flexibility in Dlx5/6+/- mice remains to be evaluated.

We hypothesize that development of modulators to selectively increasing PV+ interneuron activity may be useful for treatment of CIAS and that more potent, selective GluN2D modulators could be a viable strategy. Although our data do not address the importance of FSIN timing in the network, it is possible that increasing excitability of these interneurons may help them naturally integrate into the network and restore function. These questions will need to be addressed in future studies that include chronic treatment *in vivo* assessments in PV deficit models.

## Supporting information

S1 Table Full list of genes in RNAseq dataset (TPM, DESeq results). (XLSX)

S2 Table Murine electrophysiology dataset. (XLSX)
